# Hospital Meetings

**Published:** 1901-04-20

**Authors:** 


### HOSPITAL MEETINGS.

### MILLER HOSPITAL AND ROYAL KENT DISPENSARY.

The annual general meeting of the Governors of the Miller Hospital and Royal Kent Dispensary was held on Thursday evening, March 28th, in the board-room at the Dispensary, Greenwich Road, Mr. Edgar Sydney presiding. The accounts for the year 1899, which were presented, showed that the total ordinary income for the period amounted to £3,027, and the extraordinary income was £1,250, being the amount of two legacies. The total ordinary expenditure had been £3,755; there was no extra-ordinary expenditure, but investments figured at £1,010. The balance of expenditure over income, therefore, amounted to £488.

Mr. J. J. Vezey, F.R.M.S., presented the 118th annual report, in the absence, through a domestic affliction, of the secretary (Mr. James Marks). The report stated that the number of patients treated had totalled 16,841, the in-patients being 309; accidents, 6,252; out-patients, 7,456; patients visited, 715; midwifery cases, 497; and dental patients, 1,612. The estimated number of attendances by patients was 68,752, whilst the average number of beds constantly occupied worked out at 22·61, although there are only 25 beds in the hospital. In addition to the legacies (referred to above) the income had benefited by £550 from the Prince of Wales's Fund, by £268 from the Metropolitan Sunday Fund, and by £81 from the Saturday Fund. The committee deeply regretted the death of Mr. Thomas Moore, F.R.C.S., who had held the post of senior surgeon to the institution from its opening in 1885.

The Chairman having briefly dealt with the salient features of the report, moved its adoption.

The resolution was seconded by Dr. Hart, and unanimously agreed to.

The election of twenty-five Governors to be members of the General Committee for the ensuing year concluded the proceedings.

